# The APRIL paradox in normal versus malignant B cell biology

**DOI:** 10.1038/cddis.2016.183

**Published:** 2016-06-23

**Authors:** M H A van Attekum, A P Kater, E Eldering

**Affiliations:** 1 Department of Hematology, Academic Medical Center, University of Amsterdam, Meibergdreef 9, 1105 AZ, Amsterdam; 2 Department of Experimental Immunology, Academic Medical Center, University of Amsterdam, Meibergdreef 9, 1105 AZ, Amsterdam; 3 Lymphoma and Myeloma Center Amsterdam (LYMMCARE), Academic Medical Center, University of Amsterdam, Meibergdreef 9, 1105 AZ, Amsterdam

Chronic lymphocytic leukemia (CLL) is a classic example of a malignancy that engages in interactions with bystander cells such as T cells, stromal cells, and monocyte derived cells (MDCs) to provide itself with essential survival and proliferative signals. The important role of MDCs in the lymph node has recently been highlighted by the observation that their depletion in the T-cell leukemia/lymphoma protein 1A (TCL1) CLL mouse model by using clodronate-containing liposomes results in a better survival.^1^ Within the microenvironment, several factors can contribute to CLL protective effects, and among these a significant role has been attributed to Tumor Necrosis Factor-family members CD40L, B-cell activating factor (BAFF), and A proliferation inducing ligand (APRIL).^2^ The effects of T cell factor CD40L on both non-malignant and CLL B cells are well established; CD40L has been shown to activate both non-malignant and CLL B cells via nuclear factor kappa-light-chain-enhancer of activated B cells (NF-*κ*B) activation, thereby inducing a survival advantage via upregulation of several B-cell lymphoma 2 family members. CD40L can furthermore lead to B cell receptor-independent proliferation in both malignant and non-malignant cells, and induces class-switch recombination in non-malignant B cells (reviewed by Elgueta *et al.*^3^).

APRIL and BAFF are produced by MDCs and can bind to their cognate receptors Transmembrane activator and CAML interactor (TACI) and B-cell maturation antigen (BCMA) on target cells. In addition, BAFF can bind to a third receptor called BAFF-R.^2^ In both B cell physiology and pathology, APRIL and BAFF have been ascribed roles equally important as CD40L: both APRIL and BAFF have been reported to be responsible for the maintenance of plasma cells^4, 5^ and can induce class-switch recombination.^2^ BAFF in addition activates NF-*κ*B in healthy B cells, and is involved in mature B cell survival, whereas APRIL is not. Moreover, BAFF is critical for B cell maturation, as both BAFF and BAFF-R knockout mice lack mature B cells, an effect that is not observed in APRIL knockout mice (reviewed by Mackay and Schneider^2^).

In the context of CLL, we have previously found that overexpression of APRIL in the TCL1 mouse model accelerated disease progression.^6^ Furthermore APRIL was present in CLL lymph nodes as shown in initial experiments using quantitative PCR and immunohistochemistry. Moreover, serum APRIL levels correlate with worse prognosis in CLL patients, and these effects have been attributed to NF-*κ*B-mediated induction of CLL cell survival.^7^ These observations suggest that APRIL has an important role in CLL cell survival, but confusingly other groups were unable to recapitulate the survival effect *in vitro* using recombinant APRIL.^8, 9^ In view of this growing controversy, in a recent *Cell Death Discovery* report, we used several complementary approaches to study the role of APRIL in (MDC-mediated) CLL cell survival and proliferation.^10^

We applied a novel APRIL overexpressing system that mimics the widely applied system of CD40L stimulation, by overexpressing a fusion protein of extracellular APRIL with either the transmembrane portion of CD40L or its natural fusion partner TNF-related weak inducer of apoptosis (forming TWE-PRIL) in NIH-3T3 cells. These systems were compared with the effects of soluble APRIL produced by HEK-293 cells or recombinantly. After verifying signaling capacity using APRIL reporter cells, we analyzed direct survival effects of APRIL on CLL cells. Although CLL cells expressed both APRIL receptors TACI and BCMA, surprisingly we found no survival induction. Second, inhibition of APRIL using a TACI decoy receptor did not reduce *in vitro* macrophage-mediated survival, although these macrophages do express APRIL. We quantified their APRIL production capacity, and found it to be less than 3.13 ng/ml. In line with these negative results, APRIL stimulation did not induce canonical or non-canonical NF-*κ*B signaling, nor enhanced proliferation of CLL cells either alone or in combination with other stimuli.^10^

These observations pose a seeming paradox to APRIL's reported roles in healthy B cells, but this might be solved by looking at the developmental stage of the B cell (see Figure 1). It has been published that APRIL is able to induce survival in plasma cells,^4^ yet no survival effects was found for other developmental stages.^4^ Similarly, APRIL contributes to naive B cell proliferation, but this was not found in other B cell maturation phases.^5^ With respect to CLL cells, the effects of APRIL might overlap with its effects on precursor cell from which the CLL cell is derived. Depending on the IgV_H_ mutation status, these precursor cells have been described to be memory B cells or B1 cells.^11^ Within these precursor cells, no induction of survival was found^4^ and effects on proliferation, or NF-*κ*B activation effects have not been reported. Possibly, these precursor cells have activated a cellular program that lacks APRIL responsiveness. If true, it becomes quite plausible that CLL cells derived from these cells likewise are not affected by APRIL. In accordance with this line of reasoning, in multiple myeloma cells that are derived from an APRIL-dependent B cell stage (APRIL induces a strong survival effect).^12^

The noted absence of NF-*κ*B activation after APRIL stimulation can be contrasted to the prominent role that BAFF plays in this light. This part of the puzzle can be explained by the fact that the non-canonical pathway can exclusively be activated via the BAFF-R,^2^ to which APRIL cannot bind. Although activation of TACI and BCMA can under certain circumstances result in canonical signaling,^13^ this signaling might be dependent on the mode of activation of the receptor. Interestingly, as it has been shown that BAFF-mediated B cell survival is dependent on non-canonical signaling,^14^ the lack of APRIL effects on CLL survival could be explained by these data.

Nevertheless, other reports^7^ do suggest a direct survival effect on CLL cells when using recombinant APRIL at a concentration of 500 ng/ml. As pointed out above, the APRIL producing capacity of macrophages is apparently >100-fold lower than this. The observed effects using high APRIL concentrations might therefore be supra-physiological. Still, the enhanced disease progression observed in APRIL overexpressing TCL1 mice occurs at APRIL concentrations comparable to our human *in vitro* system, and clearly suggests that in the *in vivo* context APRIL contributes to CLL progression. A possible explanation for the apparent contrast between *in vivo* murine and *in vitro* human data might be that the role of APRIL in CLL pathogenesis might be indirect, via other cells. In a recent report, increased IL-10 production by regulatory B10 cells after stimulation of APRIL receptor TACI by recombinant BAFF was found.^15^ This increased IL-10 production could in turn result in immune suppression, thereby contributing to the immune evasion of malignant CLL cells.

We propose that absence of direct APRIL effects on CLL cells conceivably reflects that they have switched on a cellular program that derives from their non-malignant precursor cells, that neither respond to APRIL. The effects of APRIL could, however, be mediated via other cells, such as IL-10 producing B10 cells that act via indirect mechanisms on CLL cells.

## Figures and Tables

**Figure 1:**
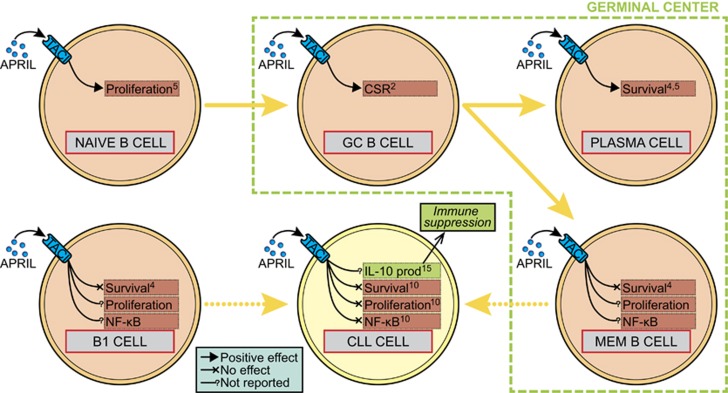
APRIL effects at different stages of B cell development and in CLL. Depending on the developmental stage of B cells, APRIL stimulation results in different outcomes. Although APRIL induces the survival of plasma cells for instance, it has no effect on survival in other stages. As CLL cells are derived from precursor B cells – namely memory B cells or B1 cells – that are unaffected by APRIL with respect to survival, proliferation or NF-*κ*B activation, the overlap in differentiation program could explain the absence of direct effects on CLL cells that we have reported in *Cell Death and Discovery*.^10^ It can, however, not be excluded that APRIL exerts its effect on CLL cells indirectly via other cell types. Stimulation of TACI in B1 cells by BAFF has recently been shown to induce IL-10 production in these cells, which could result in immune-suppressive signaling, thereby mitigating cytotoxic T-cell responses to the malignant cells. Numbers in boxes denote references. Arrows and lines denote positive effect, no effect or no reported effect of APRIL. CSR, class-switch recombination; GC, germinal center; MEM, memory; prod, production
